# 2,4-Bis(2-fluoro­phen­yl)-1-methyl-3-aza­bicyclo­[3.3.1]nonan-9-one

**DOI:** 10.1107/S1600536809053677

**Published:** 2009-12-19

**Authors:** P. Parthiban, V. Ramkumar, Yeon Tae Jeong

**Affiliations:** a Division of Image Science and Information Engineering, Pukyong National University, Busan 608 739, Republic of Korea; b Department of Chemistry, IIT Madras, Chennai, TamilNadu, India

## Abstract

The crystal structure of the title compound, C_21_H_21_F_2_NO, shows that the compound exists in a twin-chair conformation with an equatorial orientation of the *ortho*-fluoro­phenyl groups on either side of the secondary amino group. The title compound is a 1-methyl­ated analog of 2,4-bis­(2-fluoro­phen­yl)-3-aza­bicyclo­[3.3.1]nonan-9-one; the two compound both exhibit the same stereochemistry but the orientation of the *ortho*-fluoro­phenyl rings differs slightly. In the title compound, the rings are orientated at a dihedral angle of 36.70 (3)° with respect to one another, whereas in the non-methyl analog, the angle is 25.68 (4)°. The crystal structure of the title compound is stabilized by an inter­molecular N—H⋯π inter­action and a weak C—H⋯F inter­action.

## Related literature

For the synthesis and biological activities of 3-aza­bicyclo­[3.3.1]nonan-9-ones, see: Parthiban, Aridoss *et al.* (2009 ▶); Hardick *et al.* (1996 ▶); Jeyaraman & Avila (1981 ▶). For the structure of the non-methyl­ated analog of the title compound, see: Parthiban Ramkumar & Jeong (2009 ▶). For puckering and asymmetry parameters, see: Cremer & Pople (1975 ▶); Nardelli (1983 ▶).
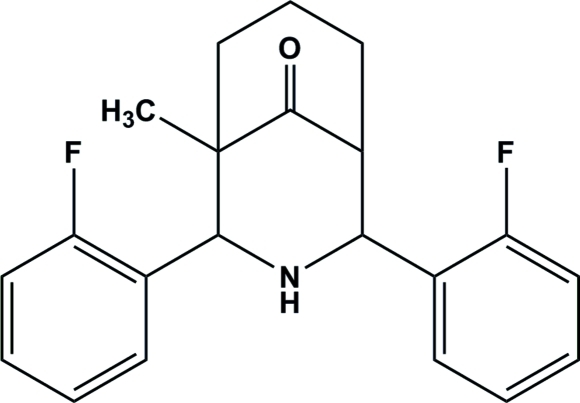


## Experimental

### 

#### Crystal data

C_21_H_21_F_2_NO
*M* *_r_* = 341.39
Triclinic, 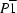

*a* = 7.8481 (3) Å
*b* = 10.5417 (4) Å
*c* = 10.9333 (4) Å
α = 76.196 (2)°
β = 80.026 (2)°
γ = 86.014 (2)°
*V* = 864.76 (6) Å^3^
*Z* = 2
Mo *K*α radiation
μ = 0.10 mm^−1^
*T* = 298 K
0.25 × 0.22 × 0.15 mm

#### Data collection

Bruker APEXII CCD area-detector diffractometer
Absorption correction: multi-scan (*SADABS*; Bruker, 1999 ▶) *T*_min_ = 0.977, *T*_max_ = 0.986
11190 measured reflections
3936 independent reflections
2176 reflections with *I* > 2σ(*I*)
*R*_int_ = 0.029

#### Refinement

*R*[*F* ^2^ > 2σ(*F* ^2^)] = 0.054
*wR*(*F* ^2^) = 0.187
*S* = 0.91
3936 reflections
231 parameters
H atoms treated by a mixture of independent and constrained refinement
Δρ_max_ = 0.16 e Å^−3^
Δρ_min_ = −0.19 e Å^−3^

### 

Data collection: *APEX2* (Bruker, 2004 ▶); cell refinement: *APEX2* and *SAINT-Plus* (Bruker, 2004 ▶); data reduction: *SAINT-Plus* and *XPREP* (Bruker, 2004 ▶); program(s) used to solve structure: *SHELXS97* (Sheldrick, 2008 ▶); program(s) used to refine structure: *SHELXL97* (Sheldrick, 2008 ▶); molecular graphics: *ORTEP-3* (Farrugia, 1997 ▶) and *Mercury* (Macrae *et al.*, 2006 ▶); software used to prepare material for publication: *SHELXL97*.

## Supplementary Material

Crystal structure: contains datablocks global, I. DOI: 10.1107/S1600536809053677/zl2256sup1.cif

Structure factors: contains datablocks I. DOI: 10.1107/S1600536809053677/zl2256Isup2.hkl

Additional supplementary materials:  crystallographic information; 3D view; checkCIF report

## Figures and Tables

**Table 1:** Hydrogen-bond geometry (Å, °)

*D*—H⋯*A*	*D*—H	H⋯*A*	*D*⋯*A*	*D*—H⋯*A*
N1—H1⋯*Cg*1^i^	0.911 (15)	2.744 (2)	3.648 (2)	171.6 (19)
C4—H4⋯F1^ii^	0.98	2.59	3.531 (3)	162

Symmetry codes: (i) 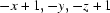; (ii) 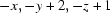. *Cg*1 is the centroid of the C9–C14 ring.

**Fig. 1.**
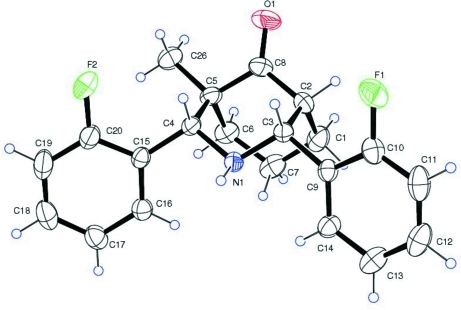
Anistropic displacement representation of the molecule with atoms represented with 30% probability ellipsoids.

**Fig. 2.**
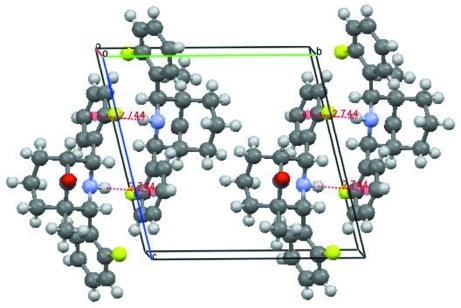
Packing diagram showing the N-H···π interaction.

**Fig. 3.**
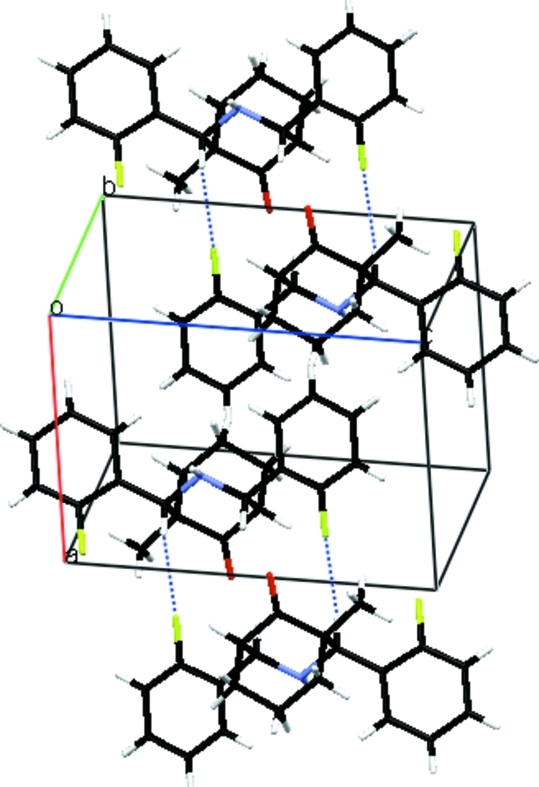
Packing diagram showing the C-H···F interaction.

## supplementary crystallographic information

### Comment

Molecules with the 3-azabicyclo[3.3.1]nonane nucleus are of great interest due to their presence in a wide variety of naturally occurring diterpenoid/norditerpenoid alkaloids and their broad-spectrum biological activities such as antimicrobial, analgesic, antogonistic, anti-inflammatory, local anesthetic hypotensive activity and so on (Parthiban, Aridoss *et al.* 2009; Hardick *et al.* 1996; Jeyaraman & Avila, 1981). Hence, the synthesis of new molecules with the 3-azabicyclo[3.3.1]nonane nucleus and their stereochemical investigation are of interest in the field of medicinal chemistry. Also, the stereochemistry of the synthesized molecules is a major criterium for their biological response. Hence, it is important to establish the stereochemistry of the bio-active molecules. As a consequence, the present study was undertaken to examine the configuration and conformation of the synthesized title compound.

The study of asymmetry parameters, ring puckering parameters, torsion angles and least-square planes calculated for the title compound shows that the bicycle exist in a twin-chair conformation. Of the chairs, the piperidine ring exists in a near ideal chair conformation with a total puckering amplitude Q_T_ of 0.605 (2) Å and a phase angle θ of 179.46 (19)° (Cremer & Pople, 1975). The smallest displacement asymmetry parameters are q_2_ = 0.015 (2) and q_3_ = -0.605 (2) Å (Nardelli, 1983). In the piperidine ring C2/C3/N1/C4/C5/C8, the ring atoms N1 and C8 deviate from the C2/C3/C4/C5 plane by -0.668 (3) and 0.689 (3) Å, respectively.

According to the crystallographic analysis, the cyclohexane ring slightly deviates from the ideal chair conformation. The total puckering amplitude Q_T_ is 0.556 (3) Å and the phase angle θ is 165.5 (3)° (Cremer & Pople, 1975). The smallest displacement asymmetry parameters q_2_ and q_3_ are 0.139 (3) and -0.539 (3)Å, respectively (Nardelli, 1983). In the cyclohexane ring C5/C6/C7/C1/C2/C8, the deviation of ring atoms C7 and C8 from the C1/C2/C5/C6 plane are -0.527 (4) and 0.713 (3) Å, respectively.

Hence the title compound, C_21_H_21_F_2_NO, exists in a twin-chair conformation with equatorial orientation of the *ortho*-fluorophenyl groups on both sides of the secondary amino group on the heterocycle. The stereochemistry of the title compound resembles that of its non-methyl analog 2,4-bis(2-fluorophenyl) -3-azabicyclo[3.3.1]nonan-9-one. However, the orientation of the *ortho*-fluorophenyl rings differ slightly. In the title compound, the *ortho*-fluorophenyl rings are orientated at an angle of 36.70 (3)° with respect to one another whereas in the non-methyl analog, they are orientated at an angle of 25.68 (4)°.

Furthermore, the title compound and its non-methyl analog 2,4-bis (2-fluorophenyl)-3-azabicyclo[3.3.1]nonan-9-one posses very similar torsion angles and molecular interactions. In the title compound, the torsion angle of C8-C2-C1-C9 and C8-C6-C7-C15 are -179.99 (3) and 179.48 (4)°, respectively (in the non-methyl analog, the equivalent torsion angles are 178.66 (4) and 179.82 (3)°, respectively).

The crystal structure of the title compound is stablized by an intermolecular N-H···π and C-H···F interactions [N1-H1 interaction with the C9/C10/C11/C12/C13/C14 ring] (Table 1). The N-H···centroid distance is 2.74 (2)Å (symmetry operator for the ring: 1-x,-y,1-z). This interaction is very similar to the N-H···π interaction observed in the non-methyl analog (N1-H1A···Cg1 = 2.72 (2) Å). The C-H···F interaction exhibits an H···F distance of 2.59Å (symmetry operator for F: -x,2-y,1-z).

### Experimental

The title compound was synthesized by a modified Mannich reaction in one-pot using 0.1 mol (12.41 g/10.52 ml) *ortho*-fluorobenzaldehyde, 0.05 mol (5.61 g/6.07 ml) 2-methlycyclohexanone and 0.075 mol (5.78 g) ammonium acetate in 50 ml of absolute ethanol. The mixture was gently warmed on a hot plate at 303–308 K (30–35° C) with moderate stirring overnight. The reaction was monitored by TLC. After all starting material was used up, the crude compound was separated by filtration and washed with a 1:5 ethanol-ether mixture. X-ray diffraction quality crystals of 1-methyl-2,4-bis(2-fluorophenyl)-3-azabicyclo[3.3.1]nonan-9-one were obtained by slow evoporation from ethanol.

### Refinement

The nitrogen H atom was located in a difference Fourier map and refined isotropically. Other hydrogen atoms were fixed geometrically and allowed to ride on the parent carbon atoms with aromatic C-H = 0.93 Å, methylene C-H = 0.97 Å, methine C-H = 0.98 Å and methyl C-H = 0.96 Å . The displacement parameters were set for phenyl, methylene and aliphatic H atoms at *U*_iso_(H) = 1.2*U*_eq_(C) and for methyl H atoms at*U*_iso_(H) = 1.5*U*_eq_(C)

### Crystal data

**Table:**

C_21_H_21_F_2_NO	*Z* = 2
*M**_r_* = 341.39	*F*(000) = 360
Triclinic, *P*1	*D*_x_ = 1.311 Mg m^−^^3^
Hall symbol: -P 1	Mo *K*α radiation, λ = 0.71073 Å
*a* = 7.8481 (3) Å	Cell parameters from 2446 reflections
*b* = 10.5417 (4) Å	θ = 2.4–22.5°
*c* = 10.9333 (4) Å	µ = 0.10 mm^−^^1^
α = 76.196 (2)°	*T* = 298 K
β = 80.026 (2)°	Block, colourless
γ = 86.014 (2)°	0.25 × 0.22 × 0.15 mm
*V* = 864.76 (6) Å^3^	

### Data collection

**Table:**

Bruker APEXII CCD area-detector diffractometer	3936 independent reflections
Radiation source: fine-focus sealed tube	2176 reflections with *I* > 2σ(*I*)
graphite	*R*_int_ = 0.029
phi and ω scans	θ_max_ = 28.3°, θ_min_ = 1.9°
Absorption correction: multi-scan (*SADABS*; Bruker, 1999)	*h* = −10→10
*T*_min_ = 0.977, *T*_max_ = 0.986	*k* = −13→13
11190 measured reflections	*l* = −14→14

### Refinement

**Table:**

Refinement on *F*^2^	Primary atom site location: structure-invariant direct methods
Least-squares matrix: full	Secondary atom site location: difference Fourier map
*R*[*F*^2^ > 2σ(*F*^2^)] = 0.054	Hydrogen site location: inferred from neighbouring sites
*wR*(*F*^2^) = 0.187	H atoms treated by a mixture of independent and constrained refinement
*S* = 0.91	*w* = 1/[σ^2^(*F*_o_^2^) + (0.1*P*)^2^ + 0.2445*P*] where *P* = (*F*_o_^2^ + 2*F*_c_^2^)/3
3936 reflections	(Δ/σ)_max_ < 0.001
231 parameters	Δρ_max_ = 0.16 e Å^−^^3^
0 restraints	Δρ_min_ = −0.19 e Å^−^^3^

### Special details

**Table:**

Geometry. All esds (except the esd in the dihedral angle between two l.s. planes) are estimated using the full covariance matrix. The cell esds are taken into account individually in the estimation of esds in distances, angles and torsion angles; correlations between esds in cell parameters are only used when they are defined by crystal symmetry. An approximate (isotropic) treatment of cell esds is used for estimating esds involving l.s. planes.
Refinement. Refinement of F^2^ against ALL reflections. The weighted R-factor wR and goodness of fit S are based on F^2^, conventional R-factors R are based on F, with F set to zero for negative F^2^. The threshold expression of F^2^ > 2sigma(F^2^) is used only for calculating R-factors(gt) etc. and is not relevant to the choice of reflections for refinement. R-factors based on F^2^ are statistically about twice as large as those based on F, and R- factors based on ALL data will be even larger.

### Fractional atomic coordinates and isotropic or equivalent isotropic displacement parameters (Å^2^)

**Table:**

	*x*	*y*	*z*	*U*_iso_*/*U*_eq_	
C1	0.2444 (4)	0.5969 (3)	0.5055 (3)	0.0676 (8)	
H1A	0.1772	0.5368	0.4799	0.081*	
H1B	0.3468	0.6168	0.4412	0.081*	
C2	0.1369 (3)	0.7231 (3)	0.5091 (2)	0.0583 (7)	
H2	0.0899	0.7515	0.4291	0.070*	
C3	0.2341 (3)	0.8380 (2)	0.52767 (19)	0.0452 (5)	
H3	0.1534	0.9132	0.5279	0.054*	
C4	0.1498 (3)	0.7686 (2)	0.75818 (19)	0.0436 (5)	
H4	0.0707	0.8451	0.7535	0.052*	
C5	0.0465 (3)	0.6521 (2)	0.7466 (2)	0.0501 (6)	
C6	0.1566 (3)	0.5251 (2)	0.7448 (3)	0.0600 (7)	
H6A	0.2077	0.5013	0.8222	0.072*	
H6B	0.0799	0.4559	0.7473	0.072*	
C7	0.3003 (4)	0.5299 (3)	0.6319 (3)	0.0668 (7)	
H7A	0.3405	0.4415	0.6289	0.080*	
H7B	0.3966	0.5760	0.6440	0.080*	
C8	−0.0112 (3)	0.6929 (3)	0.6171 (2)	0.0584 (7)	
C9	0.3833 (3)	0.8758 (2)	0.41980 (19)	0.0447 (5)	
C10	0.3558 (4)	0.9528 (2)	0.3033 (2)	0.0565 (6)	
C11	0.4824 (5)	0.9830 (3)	0.1988 (2)	0.0733 (8)	
H11	0.4570	1.0351	0.1223	0.088*	
C12	0.6469 (5)	0.9350 (3)	0.2091 (3)	0.0748 (9)	
H12	0.7346	0.9525	0.1388	0.090*	
C13	0.6817 (4)	0.8612 (3)	0.3235 (3)	0.0701 (8)	
H13	0.7941	0.8302	0.3311	0.084*	
C14	0.5517 (3)	0.8322 (2)	0.4280 (2)	0.0534 (6)	
H14	0.5783	0.7823	0.5051	0.064*	
C15	0.2188 (3)	0.7452 (2)	0.88251 (19)	0.0437 (5)	
C16	0.3783 (3)	0.6841 (2)	0.8993 (2)	0.0514 (6)	
H16	0.4454	0.6548	0.8321	0.062*	
C17	0.4399 (4)	0.6658 (3)	1.0139 (2)	0.0640 (7)	
H17	0.5467	0.6239	1.0232	0.077*	
C18	0.3426 (5)	0.7099 (3)	1.1141 (2)	0.0751 (9)	
H18	0.3837	0.6975	1.1911	0.090*	
C19	0.1865 (4)	0.7716 (3)	1.1004 (2)	0.0756 (9)	
H19	0.1205	0.8022	1.1672	0.091*	
C20	0.1285 (3)	0.7877 (3)	0.9871 (2)	0.0590 (7)	
C26	−0.1093 (3)	0.6268 (3)	0.8521 (3)	0.0708 (8)	
H26A	−0.1776	0.7061	0.8512	0.106*	
H26B	−0.0706	0.5973	0.9331	0.106*	
H26C	−0.1780	0.5610	0.8387	0.106*	
F1	0.1900 (2)	0.99679 (17)	0.29190 (15)	0.0867 (6)	
F2	−0.0261 (2)	0.85258 (19)	0.97406 (16)	0.0886 (6)	
N1	0.2938 (2)	0.80018 (18)	0.65115 (15)	0.0414 (4)	
O1	−0.1609 (2)	0.6966 (2)	0.6023 (2)	0.0911 (7)	
H1	0.352 (3)	0.868 (2)	0.661 (2)	0.061 (7)*	

### Atomic displacement parameters (Å^2^)

**Table:**

	*U*^11^	*U*^22^	*U*^33^	*U*^12^	*U*^13^	*U*^23^
C1	0.0685 (18)	0.0717 (18)	0.0706 (17)	−0.0167 (14)	0.0039 (14)	−0.0388 (14)
C2	0.0515 (15)	0.0824 (18)	0.0473 (13)	−0.0085 (13)	−0.0141 (11)	−0.0209 (12)
C3	0.0426 (13)	0.0513 (14)	0.0408 (11)	0.0064 (10)	−0.0075 (9)	−0.0106 (9)
C4	0.0388 (12)	0.0488 (13)	0.0413 (11)	0.0047 (10)	−0.0027 (9)	−0.0113 (9)
C5	0.0357 (12)	0.0569 (15)	0.0564 (13)	−0.0060 (10)	−0.0016 (10)	−0.0134 (11)
C6	0.0565 (16)	0.0509 (15)	0.0720 (16)	−0.0102 (12)	−0.0044 (12)	−0.0149 (12)
C7	0.0633 (17)	0.0474 (15)	0.091 (2)	−0.0003 (12)	0.0027 (15)	−0.0300 (13)
C8	0.0416 (14)	0.0707 (17)	0.0678 (16)	−0.0074 (12)	−0.0144 (12)	−0.0196 (13)
C9	0.0522 (14)	0.0410 (12)	0.0414 (11)	0.0013 (10)	−0.0063 (10)	−0.0118 (9)
C10	0.0731 (18)	0.0460 (14)	0.0500 (14)	0.0025 (12)	−0.0139 (12)	−0.0091 (11)
C11	0.118 (3)	0.0537 (17)	0.0451 (14)	−0.0168 (17)	−0.0047 (15)	−0.0065 (11)
C12	0.094 (2)	0.0601 (18)	0.0645 (18)	−0.0223 (16)	0.0210 (16)	−0.0207 (14)
C13	0.0612 (18)	0.0632 (17)	0.0778 (19)	−0.0068 (13)	0.0133 (14)	−0.0164 (14)
C14	0.0507 (15)	0.0532 (14)	0.0514 (13)	−0.0013 (11)	−0.0020 (11)	−0.0073 (10)
C15	0.0450 (13)	0.0445 (13)	0.0392 (11)	−0.0065 (10)	0.0008 (9)	−0.0092 (9)
C16	0.0540 (15)	0.0527 (14)	0.0463 (12)	−0.0029 (11)	−0.0064 (10)	−0.0098 (10)
C17	0.0689 (18)	0.0700 (17)	0.0522 (14)	−0.0101 (14)	−0.0164 (13)	−0.0054 (12)
C18	0.100 (2)	0.083 (2)	0.0456 (14)	−0.0274 (18)	−0.0164 (15)	−0.0100 (13)
C19	0.091 (2)	0.092 (2)	0.0472 (15)	−0.0159 (18)	0.0052 (14)	−0.0301 (14)
C20	0.0582 (16)	0.0668 (17)	0.0522 (14)	−0.0039 (13)	0.0036 (11)	−0.0221 (12)
C26	0.0518 (16)	0.088 (2)	0.0667 (17)	−0.0164 (14)	0.0069 (13)	−0.0139 (14)
F1	0.0946 (13)	0.0890 (12)	0.0711 (10)	0.0197 (10)	−0.0347 (9)	0.0008 (8)
F2	0.0749 (12)	0.1137 (14)	0.0816 (12)	0.0195 (10)	0.0047 (9)	−0.0502 (10)
N1	0.0409 (10)	0.0458 (11)	0.0380 (9)	−0.0043 (8)	−0.0051 (7)	−0.0107 (8)
O1	0.0441 (12)	0.138 (2)	0.0938 (15)	−0.0129 (11)	−0.0233 (10)	−0.0196 (13)

### Geometric parameters (Å, °)

**Table:**

C1—C7	1.518 (4)	C10—F1	1.366 (3)
C1—C2	1.530 (4)	C10—C11	1.368 (4)
C1—H1A	0.9700	C11—C12	1.367 (4)
C1—H1B	0.9700	C11—H11	0.9300
C2—C8	1.498 (3)	C12—C13	1.367 (4)
C2—C3	1.546 (3)	C12—H12	0.9300
C2—H2	0.9800	C13—C14	1.383 (3)
C3—N1	1.462 (3)	C13—H13	0.9300
C3—C9	1.511 (3)	C14—H14	0.9300
C3—H3	0.9800	C15—C16	1.388 (3)
C4—N1	1.472 (3)	C15—C20	1.390 (3)
C4—C15	1.510 (3)	C16—C17	1.386 (3)
C4—C5	1.558 (3)	C16—H16	0.9300
C4—H4	0.9800	C17—C18	1.380 (4)
C5—C8	1.517 (3)	C17—H17	0.9300
C5—C26	1.519 (3)	C18—C19	1.361 (4)
C5—C6	1.545 (3)	C18—H18	0.9300
C6—C7	1.515 (3)	C19—C20	1.361 (4)
C6—H6A	0.9700	C19—H19	0.9300
C6—H6B	0.9700	C20—F2	1.363 (3)
C7—H7A	0.9700	C26—H26A	0.9600
C7—H7B	0.9700	C26—H26B	0.9600
C8—O1	1.210 (3)	C26—H26C	0.9600
C9—C14	1.380 (3)	N1—H1	0.91 (3)
C9—C10	1.380 (3)		
			
C7—C1—C2	113.9 (2)	C14—C9—C10	115.8 (2)
C7—C1—H1A	108.8	C14—C9—C3	123.27 (19)
C2—C1—H1A	108.8	C10—C9—C3	120.9 (2)
C7—C1—H1B	108.8	F1—C10—C11	118.8 (2)
C2—C1—H1B	108.8	F1—C10—C9	117.2 (2)
H1A—C1—H1B	107.7	C11—C10—C9	124.0 (3)
C8—C2—C1	108.0 (2)	C12—C11—C10	118.7 (3)
C8—C2—C3	107.75 (19)	C12—C11—H11	120.7
C1—C2—C3	115.7 (2)	C10—C11—H11	120.7
C8—C2—H2	108.4	C13—C12—C11	119.5 (3)
C1—C2—H2	108.4	C13—C12—H12	120.2
C3—C2—H2	108.4	C11—C12—H12	120.2
N1—C3—C9	111.31 (17)	C12—C13—C14	120.8 (3)
N1—C3—C2	108.96 (18)	C12—C13—H13	119.6
C9—C3—C2	110.57 (18)	C14—C13—H13	119.6
N1—C3—H3	108.6	C9—C14—C13	121.2 (2)
C9—C3—H3	108.6	C9—C14—H14	119.4
C2—C3—H3	108.6	C13—C14—H14	119.4
N1—C4—C15	109.29 (17)	C16—C15—C20	115.3 (2)
N1—C4—C5	110.94 (17)	C16—C15—C4	122.74 (18)
C15—C4—C5	113.42 (18)	C20—C15—C4	121.9 (2)
N1—C4—H4	107.7	C17—C16—C15	121.6 (2)
C15—C4—H4	107.7	C17—C16—H16	119.2
C5—C4—H4	107.7	C15—C16—H16	119.2
C8—C5—C26	110.4 (2)	C18—C17—C16	120.0 (3)
C8—C5—C6	105.7 (2)	C18—C17—H17	120.0
C26—C5—C6	110.3 (2)	C16—C17—H17	120.0
C8—C5—C4	105.82 (18)	C19—C18—C17	120.0 (2)
C26—C5—C4	110.4 (2)	C19—C18—H18	120.0
C6—C5—C4	113.98 (18)	C17—C18—H18	120.0
C7—C6—C5	116.2 (2)	C20—C19—C18	118.9 (2)
C7—C6—H6A	108.2	C20—C19—H19	120.5
C5—C6—H6A	108.2	C18—C19—H19	120.5
C7—C6—H6B	108.2	C19—C20—F2	118.1 (2)
C5—C6—H6B	108.2	C19—C20—C15	124.2 (3)
H6A—C6—H6B	107.4	F2—C20—C15	117.7 (2)
C6—C7—C1	113.1 (2)	C5—C26—H26A	109.5
C6—C7—H7A	109.0	C5—C26—H26B	109.5
C1—C7—H7A	109.0	H26A—C26—H26B	109.5
C6—C7—H7B	109.0	C5—C26—H26C	109.5
C1—C7—H7B	109.0	H26A—C26—H26C	109.5
H7A—C7—H7B	107.8	H26B—C26—H26C	109.5
O1—C8—C2	123.5 (2)	C3—N1—C4	112.26 (16)
O1—C8—C5	123.5 (2)	C3—N1—H1	108.6 (15)
C2—C8—C5	112.95 (19)	C4—N1—H1	108.9 (15)
			
C7—C1—C2—C8	−53.1 (3)	C14—C9—C10—F1	179.6 (2)
C7—C1—C2—C3	67.6 (3)	C3—C9—C10—F1	2.5 (3)
C8—C2—C3—N1	58.7 (2)	C14—C9—C10—C11	2.0 (4)
C1—C2—C3—N1	−62.2 (2)	C3—C9—C10—C11	−175.1 (2)
C8—C2—C3—C9	−178.63 (19)	F1—C10—C11—C12	−177.7 (2)
C1—C2—C3—C9	60.5 (3)	C9—C10—C11—C12	−0.2 (4)
N1—C4—C5—C8	−56.4 (2)	C10—C11—C12—C13	−1.6 (4)
C15—C4—C5—C8	−179.84 (18)	C11—C12—C13—C14	1.4 (4)
N1—C4—C5—C26	−175.92 (19)	C10—C9—C14—C13	−2.1 (3)
C15—C4—C5—C26	60.6 (3)	C3—C9—C14—C13	174.9 (2)
N1—C4—C5—C6	59.3 (2)	C12—C13—C14—C9	0.5 (4)
C15—C4—C5—C6	−64.2 (2)	N1—C4—C15—C16	−36.4 (3)
C8—C5—C6—C7	51.3 (3)	C5—C4—C15—C16	88.0 (2)
C26—C5—C6—C7	170.7 (2)	N1—C4—C15—C20	141.4 (2)
C4—C5—C6—C7	−64.5 (3)	C5—C4—C15—C20	−94.2 (3)
C5—C6—C7—C1	−44.2 (3)	C20—C15—C16—C17	1.0 (3)
C2—C1—C7—C6	44.0 (3)	C4—C15—C16—C17	178.9 (2)
C1—C2—C8—O1	−113.1 (3)	C15—C16—C17—C18	−0.6 (4)
C3—C2—C8—O1	121.3 (3)	C16—C17—C18—C19	−0.1 (4)
C1—C2—C8—C5	64.7 (3)	C17—C18—C19—C20	0.5 (4)
C3—C2—C8—C5	−60.9 (3)	C18—C19—C20—F2	−178.4 (2)
C26—C5—C8—O1	−3.9 (4)	C18—C19—C20—C15	−0.1 (4)
C6—C5—C8—O1	115.4 (3)	C16—C15—C20—C19	−0.6 (4)
C4—C5—C8—O1	−123.4 (3)	C4—C15—C20—C19	−178.6 (2)
C26—C5—C8—C2	178.3 (2)	C16—C15—C20—F2	177.7 (2)
C6—C5—C8—C2	−62.4 (3)	C4—C15—C20—F2	−0.3 (3)
C4—C5—C8—C2	58.8 (3)	C9—C3—N1—C4	177.41 (17)
N1—C3—C9—C14	24.1 (3)	C2—C3—N1—C4	−60.4 (2)
C2—C3—C9—C14	−97.1 (2)	C15—C4—N1—C3	−173.77 (18)
N1—C3—C9—C10	−159.0 (2)	C5—C4—N1—C3	60.4 (2)
C2—C3—C9—C10	79.7 (3)		

### Hydrogen-bond geometry (Å, °)

**Table:**

Cg1 is the centroid of the C9–C14 ring.

**Table:**

*D*—H···*A*	*D*—H	H···*A*	*D*···*A*	*D*—H···*A*
N1—H1···Cg1^i^	0.911 (15)	2.744 (2)	3.648 (2)	171.6 (19)
C4—H4···F1^ii^	0.98	2.59	3.531 (3)	162

Symmetry codes: (i) −*x*+1, −*y*, −*z*+1; (ii) −*x*, −*y*+2, −*z*+1.
